# A remission spectroscopy system for *in vivo *monitoring of hemoglobin oxygen saturation in murine hepatic sinusoids, in early systemic inflammation

**DOI:** 10.1186/1476-5926-4-1

**Published:** 2005-01-12

**Authors:** Christian Wunder, Robert W Brock, Alfons Krug, Norbert Roewer, Otto Eichelbrönner

**Affiliations:** 1 Klinik und Poliklinik für Anästhesiologie, Julius-Maximilians-Universität Würzburg, Zentrum für Operative Medizin, Oberdürrbacher Strasse 6, 97080 Würzburg, Germany; 2 LEA Medizintechnik GmbH, 35394 Giessen, Germany; 3 Department of Pharmacology & Toxicology, University of Arkansas for Medical Sciences, 72205-7199 Little Rock, USA

## Abstract

**Background:**

During the early stages of systemic inflammation, the liver integrity is compromised by microcirculatory disturbances and subsequent hepatocellular injury. Little is known about the relationship between the hemoglobin oxygen saturation (HbsO_2_) in sinusoids and the hepatocellular mitochondrial redox state, in early systemic inflammation. In a murine model of early systemic inflammation, we have explored the association between the sinusoidal HbsO_2 _detected with a remission spectroscopy system and 1.) the NAD(P)H autofluorescence (an indicator of the intracellular mitochondrial redox state) and 2.) the markers of hepatocellular injury.

**Results:**

Animals submitted to 1 hour bilateral hindlimb ischemia (I) and 3 hours of reperfusion (R) (3.0 h I/R) exhibited lower HbsO_2 _values when compared with sham. Six hours I/R (1 hour bilateral hindlimb ischemia and 6 hours of reperfusion) and the continuous infusion of endothelin-1 (ET-1) further aggravated the hypoxia in HbsO_2_. The detected NAD(P)H autofluorescence correlated with the detected HbsO_2 _values and showed the same developing. Three hours I/R resulted in elevated NAD(P)H autofluorescence compared with sham animals. Animals after 6.0 h I/R and continuous infusion of ET-1 revealed higher NAD(P)H autofluorescence compared with 3.0 h I/R animals. Overall the analysed HbsO_2 _values correlated with all markers of hepatocellular injury.

**Conclusion:**

During the early stages of systemic inflammation, there is a significant decrease in hepatic sinusoidal HbsO_2_. In parallel, we detected an increasing NAD(P)H autofluorescence representing an intracellular inadequate oxygen supply. Both changes are accompanied by increasing markers of liver cell injury. Therefore, remission spectroscopy in combination with NAD(P)H autofluorescence provides information on the oxygen distribution, the metabolic state and the mitochondrial redox potential, within the mouse liver.

## Background

Hepatic microcirculatory failure is a major prerequisite for the development of hepatocellular dysfunction in a number of conditions like trauma/hemorrhage, liver transplantation and systemic inflammation. In various inflammatory states, the degree of lethal hepatocyte necrosis can be predicted from the extent of hepatic microcirculatory failure [1], possibly via alterations in the mitochondrial redox state of the liver [2,3]. Previously, our group has shown that the development of systemic inflammation was associated with a disturbance of the hepatic microcirculation, and a subsequent increase in hepatocellular damage [4,5]. The causal mechanisms are not completely understood, but accumulating evidence suggests a dysregulation of stress-inducible vasoactive mediators like endothelins, nitric oxide synthase or heme oxygenase [6]. Moreover, modifications in effector cell function may also alter the response to those mediators [7]. Hepatic microcirculatory failures during various stresses are typically characterized by alterations in the distribution of perfusion, thereby resulting in a disparity between oxygen supply and demand. This impaired nutritive blood flow, together with reduced oxygen availability, decreases cellular high-energy phosphates leading to an early hepatocellular injury and dysfunction. Studies of tissue oxygenation focusing on the relationship between microcirculatory disturbances and oxygen transport dynamics may help to better elucidate the pathophysiological mechanisms involved.

Several methods have been reported in the past couple of years directly quantifying the oxygen distribution in tissues; however, their applicability in tissues, especially in small rodents like mice, is limited due to technical reasons. For instance, microelectrodes measure tissue pO_2 _at specific points; but the technique is invasive and consumes oxygen. Electron paramagnetic resonance oximetry techniques or nuclear MRI approaches allow the detection of changes in tissue pO_2_; however, their resolution is too low [8]. A fluorescent membrane, developed by Itoh *et al*. [9] on the basis of an oxygen-quenched fluorescent dye allows the *in vivo *visualization of the tissue pO_2_. This technique allows the visualization of oxygen distribution on tissue surfaces, but this method comprised some technical limitations. The oxygen-sensitive membrane has to be used under gastight and watertight conditions during microscopy and the fluorescent membrane shows a photobleaching effect. Paxian *et al*. [10] recently demonstrated that the intravenous infusion of a special oxygen quenching dye allowed the visualization of the oxygen distribution on the liver surface using intravital videomicroscopy. The fluorescence of the dye was directly dependent on the tissue pO_2_. A disadvantage of this method, especially when used in small rodents like mice, is that it requires changing the continuous intravenous infusion rates of the dye to provide stable plasma concentrations. With mice (increasingly used as laboratory animals) there is a growing need for a method able to reliably detect tissue oxygenation or, at least, hemoglobin oxygen saturation (HbsO_2_) in capillaries of small animals.

The aim of the present study was to investigate whether the utility of a new and simple remission spectroscopy system allows reliable *in vivo *detection of liver sinusoidal HbsO_2_. In a mouse model of early systemic inflammation, we examined whether the detected changes in hepatic HbsO_2 _correlated with the established method of NAD(P)H autofluorescence and hepatocellular injury.

## Results

### Macrohemodynamics

Consistent with previous reports [4,11], mean arterial pressure (MAP) was significantly lower in animals after ischemia (I) and reperfusion (R) (3.0 h I/R and 6.0 h I/R) compared to sham animals, but remained normotensive (> 80 mmHg) throughout the study. MAP did not differ between the I/R groups. Central venous pressure was not different (data not shown).

### Blood gas analysis

The measurement of arterial blood gases carried out after the microscopy procedure showed normal oxygenation, a moderate acidosis, and adequate pCO_2 _for all groups (Table 1).

**Table 1:** Arterial blood gases.

	pO_2 _(mmHg)	pH	pCO_2 _(mmHg)
Sham	128 (46)	7.29 (0.13)	35.8 (11.3)
3.0 h I/R	123 (49)	7.27 (0.15)	36.7 (10.8)
6.0 h I/R	116 (38)	7.26 (0.17)	36.8 (12.4)
6.0 h I/R+endothelin-1	119 (46)	7.26 (0.13)	36.9 (12.9)

Data expressed as Mean (SD); n = 7 for each group

### Hepatic sinusoidal hemoglobin oxygen saturation (HbsO_2_)

**Figure 1:**
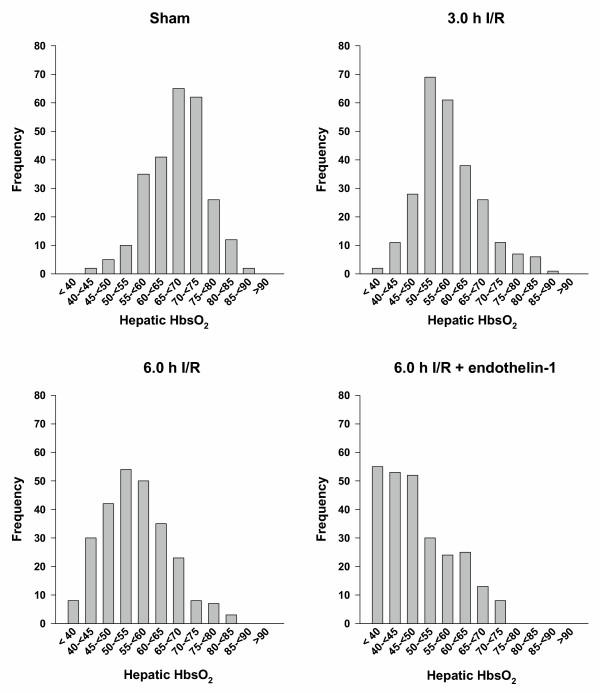
**Sinusoidal haemoglobin oxygen saturation (HbsO_2_)**. At least 35 different observation points of the left liver lobe per animal were examined. The frequency distributions of all examined HbsO_2 _values per group are shown.

Hepatic sinusoidal HbsO_2 _of the different groups are shown in Figure 1. Animals treated with 3.0 h I/R have significant lower hepatic HbsO_2 _values (56.2 (13.1)) when compared with sham (68.4 (14.1); *p *< 0.01). No statistically significant differences were observed between 3.0 h I/R and 6.0 h I/R treated animals. However, an obvious shift of hepatic HbsO_2 _towards a lower oxygenation was observed when compared with 3.0 h I/R treated animals. Animals treated with 6.0 h I/R and a continuous infusion of endothelin-1 (ET-1) showed significant reduced HbsO_2 _values (44.8 (14.7)) when compared with 3.0 h I/R treated animals (56.2 (13.2); *p *< 0.006). More than half of the measured data from these animals revealed HbsO_2 _values lower than 50%. There was no apparent difference in the local tissue hemoglobin (Hb) content detected (data not shown).

### Hepatic tissue redox status

**Figure 2:**
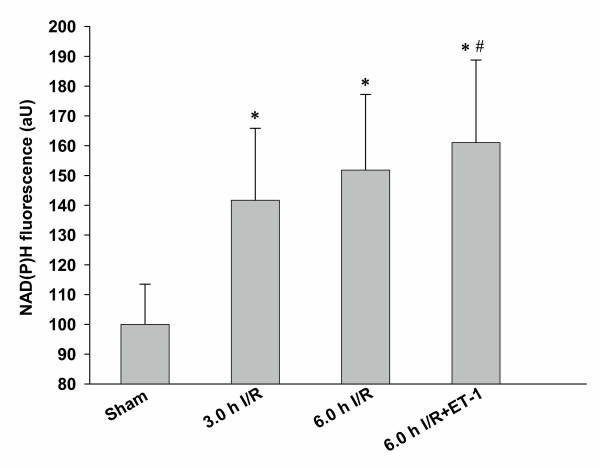
**Hepatic tissue redox status**. NAD(P)H autofluorescence, as a marker of the intracellular mitochondrial redox state, was examined using fluorescence intravital videomicroscopy with a filter set consisting of a 365 nm excitation and a 397 nm emission bandpass filter. The complete left liver lobe was systematically scanned and at least 15 different fields of view have been analysed. Fluorescence was densitometrically assessed and expressed as average intensity/liver acinus. * *p *< 0.001 vs. sham; # *p *< 0.01 vs. 3.0 h I/R; Data expressed as Mean + 2SD; n = 7 for each group.

**Figure 3:**
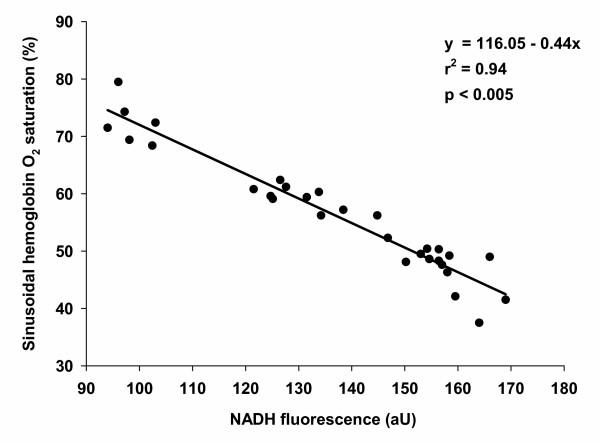
**Correlation between sinusoidal hemoglobin oxygen saturation (HbsO_2_) and tissue redox status**. The mean HbsO_2 _values significantly correlated with the corresponding NAD(P)H autofluorescence (*p *< 0.005; r^2 ^= 0.94). Data derived from 32 animals.

Animals subjected to 3.0 h I/R revealed significantly higher NAD(P)H autofluorescence (141.6 (12.8)); therefore, a significant decline in hepatic tissue oxygenation was observed when compared with sham (100.0 (6.7)) (Figure 2). Three hours I/R treated animals failed to show a significant difference in NAD(P)H autofluorescence when compared with the 6.0 h I/R treated animals. Animals treated with 6.0 h I/R and a continuous infusion of ET-1 demonstrated significantly higher NAD(P)H autofluorescence (161.1 (13.8)) when compared to the 3.0 h I/R treated animals (141.6 (12.8)). There was a highly significant correlation found between NAD(P)H autofluorescence and hepatic HbsO_2 _detected in the same animal (*p *< 0.005; r^2 ^= 0.94), as depicted in Figure 3.

### Hepatic tissue injury

**Figure 4:**
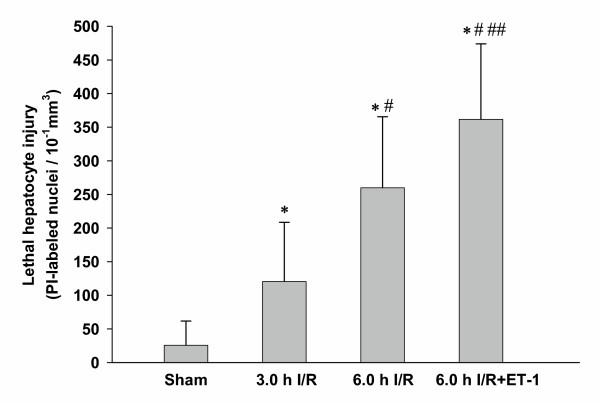
**Hepatic tissue injury**. Nuclei of lethaly injured hepatocytes were labelled *in vivo *with propidium iodide (PI). PI-labelled nuclei were quantified using fluorescence intravital videomicroscopy with a 510 to 560 nm excitation and an emission barrier filter greater than 590 nm. PI-labelled hepatocytes were expressed as number of cells/10^-1^mm^3^. * *p *< 0.001 vs. sham; # *p *< 0.001 vs. 3.0 h I/R; ## *p *< 0.01 vs. 6.0 h I/R; Data expressed as Mean + 2SD; n = 7 for each group.

**Figure 5:**
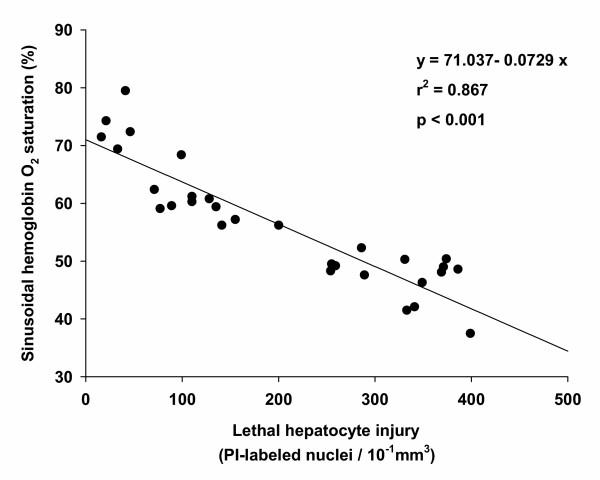
**Correlation between sinusoidal hemoglobin oxygen saturation (HbsO_2_) and lethal hepatocyte injury**. There is a significant correlation between the mean HbsO_2 _values and the corresponding amount of PI-labelled nuclei (*p *< 0.001; r^2 ^= 0.87). Data derived from 32 animals.

Serum alanine aminotransferase (ALT) and serum aspartate aminotransferase (AST) levels are summarized in Table 2. When compared with sham animals, mice treated with 3.0 h I/R exhibited significantly higher levels of ALT and AST. No significant changes between 3.0 h I/R and 6.0 h I/R animals were detectable. When compared with 3.0 h I/R, mice treated with 6.0 h I/R and a continuous infusion of ET-1 showed significant higher ALT and AST levels. The results of labelling lethally injured hepatocytes with propidium iodide (PI) are shown in Figure 4. The 3.0 h I/R treated animals exhibited a significantly increase in lethally injured hepatocytes (120.4 (44.0)) compared with sham (25.7 (17.9)), whereas the 6.0 h I/R group had a significant higher number of dead hepatocytes (260.1 (52.7)) than the 3.0 h I/R treated animals. The treatment of 6.0 h I/R animals with a continuous ET-1 infusion further elevated the degree of lethally injured hepatocytes (361.8 (56.0)) when compared to the 6.0 h I/R treated animals. Regression analysis between lethally injured hepatocytes and hepatic HbsO_2 _revealed a significant correlation (*p *< 0.001; r^2 ^= 0.86), as shown in Figure 5.

**Table 2:** Serum levels of alanine aminotransferase (ALT) and aspartate aminotransferase (AST).

	Sham	3.0 h I/R	6.0 h I/R	6.0 h I/R+endothelin-1
ALT (U/L)	50.2 (16.6)	197.0 (40.4) *	226.2 (38.5) *	261.6 (37.8) *##
AST (U/L)	177 (34)	1825 (410) *#	2551 (616) *	2856 (320) *##

Data expressed as Mean (SD); n = 7 for each group; * p < 0.001 vs. sham; # p < 0.02 vs. 6.0 h I/R; ## p < 0.01 vs. 3.0 h I/R.

## Discussion

In the present study, we demonstrate the utility of a remission spectroscopy system for the *in vivo *measurement of murine hepatic sinusoidal HbsO_2 _that showed a significant correlation with the established method of NAD(P)H autofluorescence, as well as with the extent of hepatic tissue injury.

Oximetry relies on the detection of the spectral properties of oxygenated and reduced Hb. *In vitro *bench analysis capabilities have spurred the desire to accomplish accurate *in vivo *measurement through various techniques. The 1930's and 1940's were a particularly active period for oximetry advances culminating in the development of pulse oximeters in the 1970's [12]. Remission spectroscopy is based on the same principles of those oximeters, namely because they rely on the emission of white light and measure the total intensity of the backscattered light returned from the tissue. The intensity of the backscattered light is dependant on the amount and absorbance of the Hb in the tissue under observation. Oxygenated Hb has a different absorbance from that of deoxygenated Hb. The analysis of the backscattered light spectrum allows the determination of the HbsO_2 _in the tissue. Previously, it has been shown that bilateral hindlimb I/R results in the deterioration of liver microcirculation [13]. Since the hepatic Hb content was not found to be different between groups in this study, the differences in the backscattered light spectra only represent differences in the HbsO_2_.

In the past, we have shown that bilateral hindlimb I/R results in a systemic inflammation with hepatic microcirculatory disturbances, in terms of reduced sinusoidal diameters and sinusoidal volumetric blood flow accompanied by elevated levels of sinusoidal leukocytes [4,5]. These disturbances may result in an imbalance between oxygen supply and oxygen demand. Since the spectra, extinction coefficient, and quantum yield of NADH and NADPH are the same [14,15], they are designated together as NAD(P)H – this naturally occurring fluorophore transfers electrons to oxygen by means of an electron transport chain located at the inner membrane of mitochondria [16]. Under hypoxic conditions, with no oxygen available to accept electrons from cytochrome a, intracellular NAD(P)H accumulates. Unlike the oxidized form NAD^+^, NAD(P)H is highly fluorescent [17]. Therefore, we compared the changes in NAD(P)H autofluorescence, which reflect the extent of tissue hypoxia, with that of hepatic HbsO_2 _obtained by the remission spectroscopy system under pathophysiological conditions. Whether induced by I/R or by the combination of I/R and infusion of ET-1, both analytical methods showed a decrease in hepatic oxygen supply, either as an elevation in NAD(P)H autofluorescence or as a diminution in hepatic HbsO_2_. The significant correlation between remission spectroscopy and NAD(P)H fluorescence indicates that after 3.0 h I/R, 6.0 h I/R and 6.0 h I/R+ET-1, hepatic oxygen supply was compromised. This is further emphasized by the statistical relationship found between hepatic HbsO_2 _and the extent of subsequent hepatocyte death.

Both remission spectroscopy and NAD(P)H autofluorescence provide information on the metabolic state of the murine liver. Remission spectroscopy is directly dependent on the HbsO_2 _in the sinusoids, whereas NAD(P)H autofluorescence depends upon the mitochondrial redox state and the activity of the mitochondrial electron transport chain. It was previously proposed that during systemic inflammation the NADH/NAD^+ ^redox potential may increase, and oxygen utilization may be altered [18]. The present study demonstrates a concomitant change in NAD(P)H autofluorescence and hepatic HbsO_2_. Obviously, the observed hypoxia did not occur through altered oxygen utilization, but rather through a reduced oxygen supply induced by sinusoidal microcirculatory disturbances. This corroborates our previous contention that the simultaneous use of remission spectroscopy, and that of NAD(P)H autofluorescence, provides additional information regarding the underlying pathophysiological mechanisms. That technical approach allows the correlation between disturbances in oxygen supply and those of oxygen utilization.

## Conclusions

There is a significant reduction in hepatic sinusoidal HbsO_2 _during the early stages of systemic inflammation. In parallel, we detected an increasing NAD(P)H autofluorescence representing an intracellular inadequate oxygen supply. Both changes are accompanied by increasing markers of liver cell injury. Future therapeutic interventions should focus on the amelioration of sinusoidal HbsO_2 _followed by an improvement in mitochondrial redox state. Remission spectroscopy represents a simple and reliable method for hepatic sinusoidal HbsO_2 _determination in small rodents. In combination with NAD(P)H autofluorescence, it provides information on the oxygen distribution, the metabolic state and the mitochondrial redox potential within the hepatic tissue.

## Methods

### Animals

Male C57/BL6 mice (eight to ten weeks old, weighing 23.7 (11.1) g) were used for all experiments. The experimental protocols were in compliance with the guidelines of the Committee on the Care and Use of Laboratory Animals of the Institute of Laboratory Animal Resources, National Research Council as well as those of Germany. Animals were maintained under controlled conditions (22°C, 55% humidity and 12-hour day/night cycle) with free access to tap water and a standard laboratory chow.

### Experimental protocol

Mice (n = 7, for each group) were randomly assigned to either a Sham or a hindlimb ischemia/reperfusion (I/R) group. Animals of the I/R groups were treated with 60 minutes bilateral hindlimb ischemia induced by tightening a tourniquet above the greater trochanter of each leg while under anaesthesia. Sham animals were not subjected to ischemia, but remained anaesthetized for the same period of time. Tourniquets were removed just prior to recovery from anaesthesia. The animals were awake during the 3 hours (3.0 h I/R) or the 6 hours (6.0 h I/R) reperfusion periods, and re-anaesthetized for the intravital microscopy procedure.

To further induce liver microcirculatory disturbances and contribute towards a reduction in liver oxygen supply 6.0 h I/R, mice were further randomized to a group treated with a continuous infusion of ET-1 (70 pmol/min., i.v.) starting 15 minutes prior to microscopy. This dose of ET-1 was chosen because it produced alteration in the oxygen distribution, along with derangements in the hepatic tissue perfusion [19].

### Surgical procedure

Animals received anaesthesia, by inhalation, for all procedures. As previously described [20], anaesthesia was performed using isoflurane (Forene, Abbott, Wiesbaden, Germany) in spontaneously breathing animals. The left carotid artery and the left jugular vein were cannulated under sterile conditions. The carotid artery cannula was used for the continuous measurement of systemic arterial blood pressure and heart rate, while central venous pressure was assessed via the jugular vein cannula. Throughout the experiment, normal saline was administered at a rate of 0.4 ml/hr to maintain normal mean arterial pressure. As formerly described [4], and for the realization of the intravital microscopy procedure in anaesthetized animals, a transverse subcostal incision was performed. Briefly, the ligament attachments from the liver to the diaphragm and to the abdominal wall were carefully released. For the evaluation of the hepatic microcirculation by intravital fluorescence microscopy, the animals were positioned on left lateral decubitus and the left liver lobe was exteriorized onto an adjustable stage. The liver surface was covered with a thin transparent film to avoid tissue drying and exposure to ambient oxygen. For equilibrium purposes, a pause of 10 minutes was allowed before data from microscopy and remission spectroscopy was collected. After microscopy, animals were killed by exsanguination, via the insertion of a cannula in the left femoral artery for the collection of arterial blood samples or via cardiac puncture.

### Intravital microscopy

Details of this technique have been described elsewhere [4,21]. For observations of the liver microcirculation, we used a modified inverted Zeiss microscope (Axiovert 200, Carl Zeiss, Göttingen, Germany) equipped with different lenses (Achroplan × 10 NA 0.25 / × 20 NA 0.4 / × 40 NA 0.6). The image was captured using a 2/3" charge-coupled device video camera (CV-M 300, Jai Corp., Kanagawa, Japan) and digitally recorded (JVC HM-DR10000EU D-VHS recorder) for off-line analysis. As previously described [22], NAD(P)H autofluorescence, as a marker of the mitochondrial redox state, was assessed using the 10x objective lens. The liver was examined using a filter set consisting of a 365 nm excitation and a 397 nm emission bandpass filter. NAD(P)H autofluorescence was recorded over the complete left liver lobe, allowing at least 15 different fields of view. Non-viable hepatocyte nuclei were labelled *in vivo *with an i.v. bolus of the vital dye PI (0.05 mg/100 g). As previously stated [21], PI-labelled nuclei were used to identify lethally injured hepatocytes. The fluorescent labelling of these nuclei was viewed using the 20x objective lens and a filter set with a 510 to 560 nm excitation and an emission barrier filter greater than 590 nm. Quantification of redox state and cell death was performed off-line by frame-by-frame analysis of the videotaped images using Meta Imaging Series Software (Ver. 6.1; Universal Imaging Corp., Downington, PA, USA). NAD(P)H fluorescence was densitometrically assessed and expressed as "average intensity/liver acinus". Gain, black level and enhancement settings were identical in all experiments. PI-labelled hepatocytes were expressed as number of cells/10^-1 ^mm^3^.

### Remission spectroscopy

**Figure 6:**
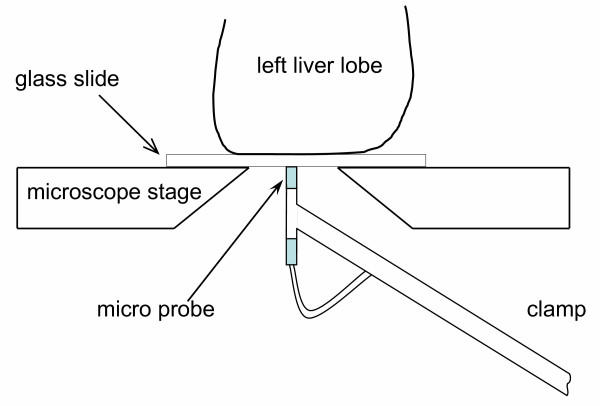
**Illustration of the experimental setup**. The flexible probe of the remission spectroscopy system was fixed on a special shaped clamp holder, which allowed the contact free scanning of the left liver lobe from the bottom side of the glass slide. The setup permitted systematic *in vivo *scanning of the liver sinusoidal HbsO_2_, without affecting the organ integrity.

Hepatic sinusoidal HbsO_2 _was measured using the remission spectroscopy system Oxygen-to-See (O2C-ATS) supplied with the micro probe VM-3 (Lea Medizintechnik GmbH, Gießen, Germany). White light was continuously emitted via one channel of the micro probe light-guide and was continuously detected via another channel (channel diameter 70 μm). The backscattered light was analyzed in steps of 1 nm (500–650 nm). Each HbsO_2 _value was defined by specific Hb spectra. The local tissue light absorbance depends on the total local tissue content of Hb. The local content of Hb was calculated from the local light absorbance and emission. The flexible VM-3 micro probe allowed the detection of oxygen saturation of the left liver lobe placed on the glass slide of the inverted microscope. A special clamping system fixed the micro probe close to the surface of the glass slide and permitted contact-free systematic scanning of the liver lobe (Figure 6). At least 35 different observation points per animal were randomly chosen and examined. Before each experiment, the white standard of the micro probe was calibrated according to the technical instructions of the manufacturer.

### Measurement of serum alanine aminotransferase (ALT) and aspartate aminotransferase (AST) levels

Blood was collected immediately after the microscopy procedure, via cardiac puncture. Blood samples were centrifuged at 6500 g, for 5 min, and the remaining serum analyzed, at 37°C, by means of standard enzymatic techniques.

### Blood gas analyses

Blood samples for blood gas analyses were collected in heparinized syringes, via the insertion of a cannula in the left femoral artery, at the end of the microscopy procedure. The samples were immediately analyzed using the automated blood gas analyzing system Radiometer ABL 700 (Radiometer Medical Aps., Bronshoj, Denmark).

### Statistical analysis

Data in text and Tables is given as: Mean (SD). Statistical differences between groups and from baseline within each group were determined by ANOVA, followed by the Tukey post-hoc test. The Kolmogorov-Smirnov test was previously used to confirm the normal distribution of data. For checking the nature and extend of the relationship between two variables linear regression analysis was performed. All figures were generated with Sigma Plot (Ver. 8.0) and statistical analyses were performed using Sigma Stat software (Ver. 2.0; SPSS Inc.; München, Germany). Differences were considered significant for *p *< 0.05.

## Authors' contributions

CW conceived the design of the study and conducted the laboratory experiments; RB drafted the manuscript and coordinated the study; AK assisted in technical questions. NR participated in design and coordination and OE participated in animal procedures and in drafting the paper. All authors approved and read the final manuscript.
